# Pharmacokinetics and safety of panitumumab in a patient with chronic kidney disease

**DOI:** 10.1007/s00280-017-3479-2

**Published:** 2017-11-23

**Authors:** L. L. Krens, J. M. Baas, H. J. Guchelaar, H. Gelderblom

**Affiliations:** 1 Department of Clinical Pharmacy and Toxicology, Leiden University Medical Center, Albinusdreef 2, 2300 RC Leiden, The Netherlands; 2 ZGT Pharmacy, Hospital Group Twente, Boerhaavelaan 63, 7555 BB Hengelo, The Netherlands; 3 Department of Medical Oncology, Leiden University Medical Center, Albinusdreef 2, 2300 RC Leiden, The Netherlands

**Keywords:** Panitumumab, Pharmacokinetics, Renal insufficiency, Colorectal cancer

## Abstract

**Purpose:**

Data on panitumumab dosing in cancer patients with renal insufficiency are lacking. Here, we report a 63-year-old metastatic colorectal cancer patient with chronic kidney injury with a glomerular filtration rate of approximately 11 mL/min.

**Methods:**

Pharmacokinetic parameters, including dose-normalized area under the curve, clearance and elimination half-life (*T*_1/2_) after the 11th and 12th infusions were estimated using trapezoidal non-compartmental methods. Data were compared to previous reported pharmacokinetic data from studies in patients with normal renal function.

**Results:**

The results show that the pharmacokinetic data in this patient with kidney failure are comparable to those in patients with adequate renal function. Moreover the treatment was well tolerated in this patient.

**Conclusion:**

This study suggests that panitumumab can be safely used in cancer patients with renal impairment without dose adjustment.

## Introduction

Panitumumab is a fully humane monoclonal antibody targeting the epidermal growth factor receptor (EGFR) and is registered for the treatment of *RAS* wild-type metastatic colorectal cancer, either alone or combined with chemotherapy. As previously discussed elsewhere, clearance of panitumumab mainly occurs by an EGFR sink. In case of saturation of all receptors, panitumumab will be cleared by immunologic mechanisms, such as complement-dependent cytotoxicity (CDC), antibody dependent cell-mediated cytotoxicity and apoptosis [1]. Therefore, theoretically renal insufficiency is not likely to influence the pharmacokinetics of panitumumab. The study of councilman et al. showed that nephrotic syndrome was associated with increased rituximab clearance, and therefore, decreased half-life. An possible explanation for the observed effect is loss of monoclonal antibody in the urine and not altered clearance [2].

The most recent summary of product characteristics (SmPc) of panitumumab states that a population pharmacokinetic analysis (among race, age, gender, hepatic function, concomitant chemotherapy and EGFR membrane-staining intensity in tumor cells) renal function does not influence the pharmacokinetics of panitumumab, however, it is not tested in patients. The only available clinical information concerns a case report showing safety and efficacy of panitumumab (combined with oxaliplatin, folic acid and 5-FU) in a hemodialysis patient [3]. However, to our knowledge, this is the first case study showing actual pharmacokinetic parameters in a patient with chronic kidney injury without dialysis (CKD).

## Subject and methods

### Case

A 63-year-old Caucasian male was diagnosed with colon cancer with hepatic metastases in November 2011. His medical history included diabetes type 2, congestive heart failure and CKD with unknown etiology. The estimated clearance according to the modification of diet in renal disease (MDRD) was 21 mL/min at this time. The primary tumor was resected because of its obstructive character. Histopathological analysis showed a poorly differentiated adenocarcinoma, *KRAS* wild type. A few weeks later, the patient started palliative chemotherapy, consisting of oxaliplatin, folic acid and 5-FU (FOLFOX). This therapy was discontinued after eight cycles since his renal function declined. After a period without treatment, he started with panitumumab in May 2013. By then, his renal function had further declined to an estimated clearance of 11 mL/min (MDRD). His weight was 106 kg. Panitumumab was dosed at the recommended full dose of 6 mg/kg diluted in 100 mL sodium chloride solution (0.9%) and administered in 60 min, without pre-medication following a standard procedure. Serum samples for pharmacokinetic analysis were collected after the 11th and 12th infusion of panitumumab and toxicity data were collected. The patient gave informed consent and the Medical Ethics Committee approved the study.

### Panitumumab sampling and measuring

Serum samples were planned at 0.5, 1, 2, 4, 8, 24 h, 4 days, and 7 days after the 11th panitumumab infusion. Before the 12th infusion (day 15) and 30 min, 1 h and 14 days later the blood samples were drawn. The samples were allowed to clot for 30 min, followed by centrifuging at 3000 rounds per minutes. The serum was transferred to a tube and stored at − 80 °C until analysis.

Panitumumab serum drug concentrations were performed by PPD laboratories (Richmond, VA, USA) using a validated immunoassay with electrochemiluminescence as described before [1].

### Pharmacokinetic parameters

Pharmacokinetic parameters were estimated by trapezoidal noncompartmental methods using MW/PHARM 3.5 of Mediware (Groningen, The Netherlands). Pharmacokinetic parameters for panitumumab i.e., area under the serum concentration–time curve (AUC) maximum observed serum concentration (*C*_max_), and minimum observed serum concentration (*C*_min_)—were determined. Half-life (*T*_1/2_), volume of distribution (*V*) and clearance (CL) were calculated.

For comparison, historical data from the summary of product characteristics (SPC) [2] and cohort 1 of Stephenson et al. were used [4]. From this study, the dose-normalized (for the dose of 6 mg per kg) AUC, clearance, elimination half-life, minimum and maximal concentrations were used. In case the value was within the reported serum level ± 1 standard deviation, the found value was considered not to be clinically relevant or clinically different.

### Toxicity

Information on toxicities were collected at baseline, just before each course, at the day of infusion and 7 days after infusion. Information on toxicities were also collected during each unplanned hospital visit or contact. Toxicities were graded using CTCAE version 1.1.

## Results

### Case

A total of 12 infusions of panitumumab were administered. The patient experienced grade 2 skin toxicity, treated with topical agents (no minocycline because of the CKD). At the beginning his condition improved significantly and he had a mixed response with regression of liver metastases and new pleural metastases. However, after the 12th cycle of panitumumab in the end of October 2013, his lesions had clearly progressed. Treatment with regorafenib was considered in November 2013, however, by then his CKD had progressed further and at that point starting dialysis seemed inevitable (mostly because of electrolyte disturbances). Considering his poor prognosis, patient declined dialysis and soon after that he was admitted to a hospice. He died a few weeks later.

### Pharmacokinetics

The maximal observed serum concentration of panitumumab was 125 μg/mL after the 11th and 12th infusion. The minimum concentration observed just before the 12th infusion was 37.0 and 48.0 μg/mL, 13 days after the 12th infusion. The reported serum concentrations were used to calculate the AUC, half-life and clearance (Table 1).

**Table 1:** Pharmacokinetic parameters of the case and historical data from SPC and the study of Stephenson et al.

Descriptive statistic	*C*_max_ (SD) (μg/mL)	*C*_min_ (SD) (μg/mL)	AUC (SD) (μg day/mL)	*T*_1/2_ (SD) (days)	CL (SD) (mL/day/kg)	*V* (SD) (L)
Cohort 1 first dose 6 mg/kg (2 weekly) [5]	152 (29.3)	18.1 (8.6)	744 (195)	5.28 (1.90)	8.21 (3.79)	
Cohort 1 first dose 6 mg/kg (2 weekly) [5]	232 (71.2)	46.6 (16.9)	1310 (375)	9.08 (3.61)	4.96 (1.49)	
SPC 6 mg/kg (2 weekly)	213 (59)	39 (14)	1306 (374)	7.5	4.9	
Case 11th infusion	125.0	37.0	1555	9.80	3.4	5.6
Case 12th infusion	125.0	48.8	1752	10.3	3.8	5.4

*SD* standard deviation

**Fig. 1:**
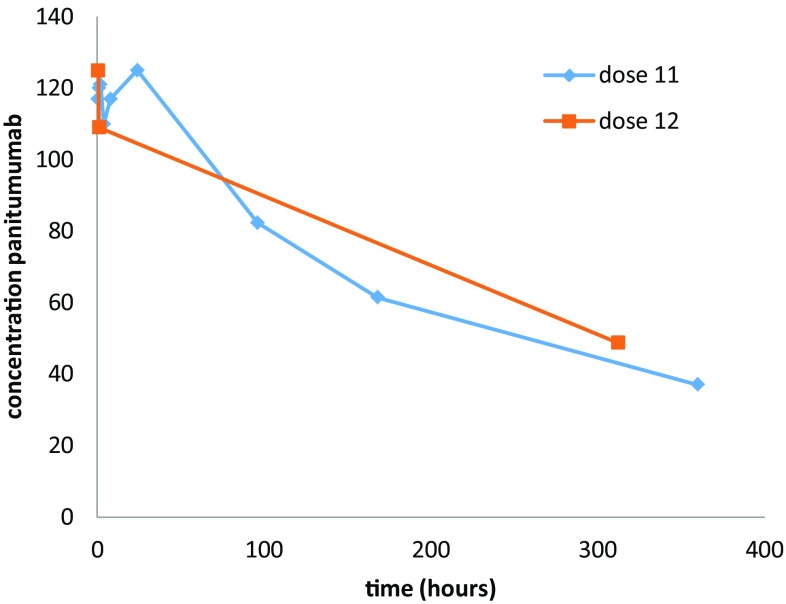
Time curve of panitumumab concentration following 1 h infusion of 616 mg of panitumumab in a patient with a glomerular filtration rate (MDRD) of approximately 11 mL per minute

In Table 1, an overview of the pharmacokinetic parameters of panitumumab in study populations with normal renal functions and this case is shown. In this table, the pharmacokinetics of the 11th and 12th infusion of 6 mg/kg in the Stephensons cohort and data from the SPC are depicted and used for comparison. In Fig. 1 the time concentration curve after the first and second infusion of panitumumab are depicted.

In this case, the calculated AUC was 1555 and 1752 μg day/mL after the 12th infusion. The calculated clearance was 3.4 and 3.8 mL/day/kg and the half-life was 9.3 and 10.8 days, respectively, after the 11th and 12th infusion. A comment should be made regarding the calculated half-life after the 12th infusion. This half-life may be biased due to limited sampling (at 30 min, 1 h and 14 days) because the distribution phase may not be terminated after one hour.

Compared to the historical data, the maximal concentration measured in our case was lower as compared to the reported maximum concentration in the SPC and the Stephenson’s cohort. Furthermore, the AUC calculated after the 12th infusion was higher in our case compared to the historical data. The AUC calculated after the 11th infusion was within the earlier reported mean and standard deviation. The minimum concentration, half-life and clearance calculated in this study were all comparable to the historical data of the cohorts and SmPC.

## Discussion

Until now, no clinical studies have been conducted to examine the pharmacokinetics of panitumumab in patients with renal impairment. Previously, we have reported a similar case study with cetuximab in CKD [5] and hereby report one on the pharmacokinetics of panitumumab in a patient with CKD. During the registration of panitumumab, a population pharmacokinetic analysis was performed to explore the potential effects of selected covariates on panitumumab pharmacokinetics. These theoretical results showed that renal function had no apparent impact on the pharmacokinetics of panitumumab. However, recommendations on dosing in patients with kidney disease are lacking. Knowledge of the precise impact of CKD on panitumumab pharmacokinetics is highly relevant as the percentage of cancer patients over 75 years is expected to increase disproportionally [6] and glomerular filtration rate naturally declines during life [7]. In addition, most patients have previously been treated with oxaliplatin which may also negatively influence renal function. Clearly, CKD is not uncommon in colorectal cancer patients.

The pharmacokinetic parameters AUC and maximum concentrations of our case are different compared to earlier reported data [4]. The pharmacokinetic parameters, minimum concentration, clearance and half-life were similar to the results reported from population without renal failure. It is important to mention that all the reported half lives in the cohorts and in the SPC are low compared to the half-life reported in the study of Ma et al. [8]. They reported a half-life of 18.3 days for panitumumab. Thus, the here reported half lives of panitumumab (case and included studies) may be overestimated due to non-linear elimination shape.

The maximum concentration reported in our patient appeared to be lower. The weight of this patient, however, was 106 kg and the BMI was 30. In obese, the total blood volume is increased; this increase could be an explanation for the lower maximum concentrations of panitumumab [9].

The AUC after the 11th infusion was comparable with historical data. The AUC after the 12th infusion was slightly higher than the reported AUC’s in former studies in patients without renal failure. The AUC during the 11th and 12th infusion were estimated in steady state so this could be due to inter-patient variability. It is unlikely to be caused by the decreased renal function in this patient because clearance of panitumumab occurs extensively by the EGFR sink and the reticuloendothelial system. Another factor which may influence the clearance is the tumor burden and the antigen density of the tumor. As a consequence a lower tumor burden or antigen density may lead to reduced clearance and thus a higher AUC.

Besides the slightly higher AUC after the 12th infusion, the pharmacokinetic parameters are in line with population without chronic kidney disease. Furthermore, during treatment, no additional toxicity was noted, except the expected skin toxicity commonly reported in EGFR antibody treatment. The absence of an effect of the renal function on the pharmacokinetics was as expected and in line with other studies. Due to their molecular size, monoclonal antibodies are not excreted by the urine. For panitumumab and other monoclonal antibodies, population pharmacokinetic studies used models to study the effect of the renal clearance as a covariate and did not find an effect on clearance [10]. In this study, we validated the lack of effect in a patient.

In conclusion, the pharmacokinetics of panitumumab between patients with decreased renal functions and patients with normal renal function seems similar.
